# Photo-Orientation of Liquid Crystals on Azo Dye-Containing Polymers

**DOI:** 10.3390/polym14010159

**Published:** 2021-12-31

**Authors:** István Jánossy, Tibor Tóth-Katona

**Affiliations:** Institute for Solid State Physics and Optics, Wigner Research Centre for Physics, P.O. Box 49, H-1525 Budapest, Hungary; janossy.istvan@wigner.hu

**Keywords:** nematic–polymer interface, photoalignment, optical sensors, optical actuators

## Abstract

In this communication, we summarise our results related to light-induced orientational phenomena at liquid crystal–polymer interfaces. We investigated photoalignment for various nematics at the interface with the photosensitive polymer layer polymethyl methacrilate functionalised with azo dye Disperse Red 1. It was found that the efficiency of photoalignment exhibits marked differences depending on the structure of the rigid core of the liquid crystal molecules. It was demonstrated that the photo-orientation process is also significantly affected by the type of mesophase in which irradiation is carried out. The observations highlight the importance of the mutual influence of the polymer and the liquid crystal in light-induced processes.

## 1. Introduction

Azo dye-containing polymers are widely used for different purposes in optics. In 1984, Todorov et al. demonstrated the feasibility of using azo dye-doped polyvinyl alcohol for holographic purposes [1]. Since then, numerous works have been carried out in connection with this subject (for a review, see, e.g., [2]). The possibility of generating surface reliefs on polymer surfaces with light has also attracted considerable interest [3,4]. Moreover, azo-containing polymers can be used as non-linear optical systems [5,6,7].

In this communication, we discuss our investigations related to the photoalignment of liquid crystals on photosensitive azo-containing polymer films. The possibility of orienting liquid crystal layers by means of photochemical processes was first demonstrated in the early 1990s. Since then, a great amount of research has been accomplished related to the subject and several excellent reviews have been published—see, e.g., [8,9,10,11,12].

In an important part of the investigations, photo-orientation was realised with the help of azo compounds, exploiting light-induced *trans-cis* isomerisation. With this method, one can switch the liquid crystal configuration between planar and homeotropic alignment [13,14], or it is possible to control the azimuthal orientation of a nematic liquid crystal on a photosensitive polymer substrate [15,16,17]. In the *reversible* photo-orientation, the surface alignment of the liquid crystal is governed by the polarisation direction of the control light beam. Upon photo-excitation, azo dyes undergo a *trans–cis–trans* cycle that can result in a change of their alignment within the polymer network. Under the influence of polarised irradiation, the azo dye molecules are selectively excited with respect to their orientation, and consequently, the orientational distribution becomes anisotropic. The anisotropic distribution created in this way contributes to the anchoring energy of the liquid crystal in contact with the photosensitive plate. The “easy axis”, due to the redistribution of the dye molecules, is in most cases as perpendicular to the polarisation of the pump beam.

The purpose of the present communication was to show that the efficiency of photo-orientation depends, in addition to the composition of the photosensitive polymer film, on the structure of the liquid crystal as well. To verify this fact, we compared a series of nematic liquid crystals with various structures, applying the same photosensitive layer (Section 3). Significant differences were found in both the induced reorientation and the dynamics of the relaxation process for liquid crystals with different rigid core compositions. We highlight the role of director gliding in the relaxation process. In addition, we revealed that photo-orientation is sensitive to the state of the liquid crystalline compound. It was found that the same irradiation caused different reorientations when it was carried out in the smectic, nematic or isotropic phase of the compounds (Section 4). We present a simple model to explain these observations, in which the interaction of the azo dyes with the liquid crystal at the interface plays an essential role. Theoretical curves based on the model show reasonable quantitative agreement with the experimental data.

## 2. Experimental and Materials

**Figure 1:**
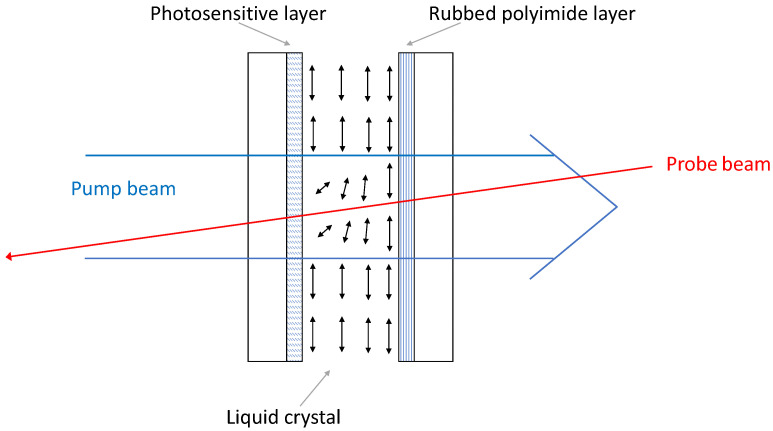
Typical cell and experimental setup used for the study of photoalignment. The liquid crystal director is reoriented on the photosensitive plate by a pump beam and the resulting distortion is measured by a weak probe beam.

A standard cell used for the investigation of photo-orientation is shown in Figure 1. In the cells, the liquid crystal was sandwiched between two plane-parallel plates. The reference substrate is typically a traditional rubbed polyimide layer that ensures the planar surface orientation of the liquid crystal on it, with a small pretilt of $\theta_{0}∼1^{{}^{\circ}}$ [18]. The photosensitive substrate contains azo dyes. In order to achieve a good planar orientation at the start of the experiment, the photosensitive plate was either irradiated with polarised light prior to filling the cell with the liquid crystal, or the cell was filled in the isotropic phase and cooled down to the liquid-crystalline phase in the presence of a magnetic field.

The reorientation was induced by a laser beam (pump beam) with wavelength set in the range of the absorption band of the azo dye. The pump beam entered the cell from the photosensitive face (direct geometry) and reoriented the liquid crystal within the beam area, as indicated in Figure 1. To measure the induced azimuthal and zenithal reorientations, a weak probe beam was applied with the wavelength lying outside the absorption band (usually a He–Ne laser beam, λ=633 nm). The probe beam entered the cell from the reference side and was focussed on the centre of the pump beam. In order to determine the azimuthal reorientation, the polarisation direction of the probe beam was measured behind the sample. The change in the phase-shift between the ordinary and extraordinary components of the probe beam was also measured, giving information of a possible zenithal reorientation during photoalignment. Details of the setup can be found in [19].

The thickness of the cell gap was in the order of 10 μm. The temperature was regulated with a precision of 0.1 °C.

**Figure 2:**
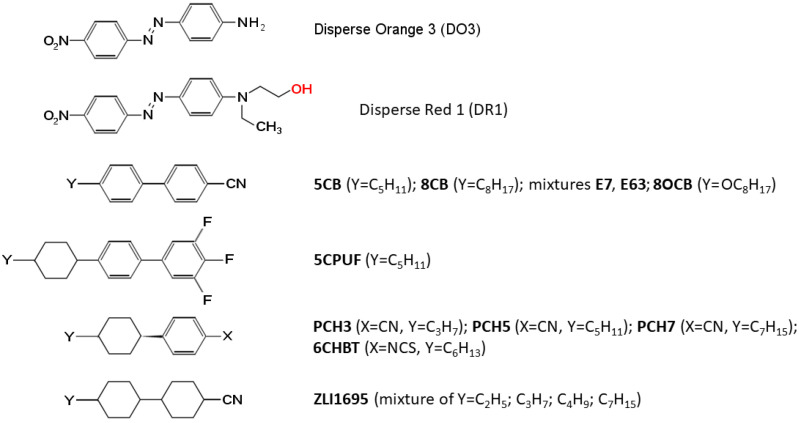
Molecular structure of the azo dyes Disperse Orange 3 (which was physically mixed with the polyimide polymer) and Disperse Red 1 (which was chemically attached to the PMMA main chain—with the reactive site labelled with red), as well as that of liquid crystals selected for the photo-orientation experiments.

For the study of the influence of the liquid crystalline molecular structure on the photo-orientation process pDR1 (*polymethyl methacrilate* (PMMA) functionalised with azo dye *Disperse Red 1* (Figure 2)) was used—see [19] for the molecular structure of the polymer. The synthetic route of pDR1 is presented, e.g., in Ref. [20]. The preparation procedure of the pDR1 layer is described in Ref. [21].

Nematic liquid crystals (LCs) having biphenyl, phenylcyclohexane, or bicyclohexane in the rigid core were selected for the photoalignment experiments—see Figure 2 for the molecular structures. The LC compounds with a biphenyl core were *4-cyano-4’-penthylbiphenyl* (5CB), *4-cyano-4’-n-octylbiphenyl* (8CB), *4-cyano-4’-n-octyloxybiphenyl* (8OCB), *3,4,5-trifluoro-4’-(4-pentyl-cyclohexyl)biphenyl* (5CPUF), as well as mixtures E7 and E63 (both from Merck). We note that the three fluorene atoms at 3,4,5 positions in 5CPUF almost “invert” the molecular electrostatic surface potential (MESP) [22] of the attached phenyl ring compared to the case of cyano-biphenyls (5CB, E7, E63) (as can be seen in [19]). LCs with phenylcyclohexane core were *1-(trans-4-hexylcyclohexyl)-4-isothiocyanatobenzene* (6CHBT) [19] and some members of the PCH homologous series (PCH3, PCH5 and PCH7) [23], while LC with bicyclohexane core was the mixture ZLI1695 [23].

Another method for preparing photosensitive layers is to physically include some non-reactive azo dyes into the polymer network. In this procedure, a polymer is dissolved into an organic solvent together with a few percent of the azo dye. The solution is spin-coated onto the substrate, forming a few hundred nm-thick film. In the film, the dye molecules do not form chemical bonds with the polymer backbone; rather, they occupy some space surrounded by the network of chains. In the case of reorientation measurements in different phases of the liquid crystal (Section 4), we used polyimide as a polymer and the push-pull azo dye Disperse Orange 3 as a dopant. Polyimide was chosen because it has a high glass transition temperature (>$340{\;}^{{}^{\circ}}$C). As it is difficult to find appropriate solvent for polyimide, the photosensitive substrate was coated with a saturated solution of the azo dye Disperse Orange 3 in polyamic acid solution and subsequently heat treated at approximately $200{\;}^{{}^{\circ}}$C to complete the imidisation.

## 3. Photoalignment with Different Nematics

Experiments have revealed three distinct processes in these systems: azimuthal photoalignment (in-plane with the photosensitive layer), zenithal (out-of-plane to the layer) photoalignment, and thermally induced reorientation.

**Figure 3:**
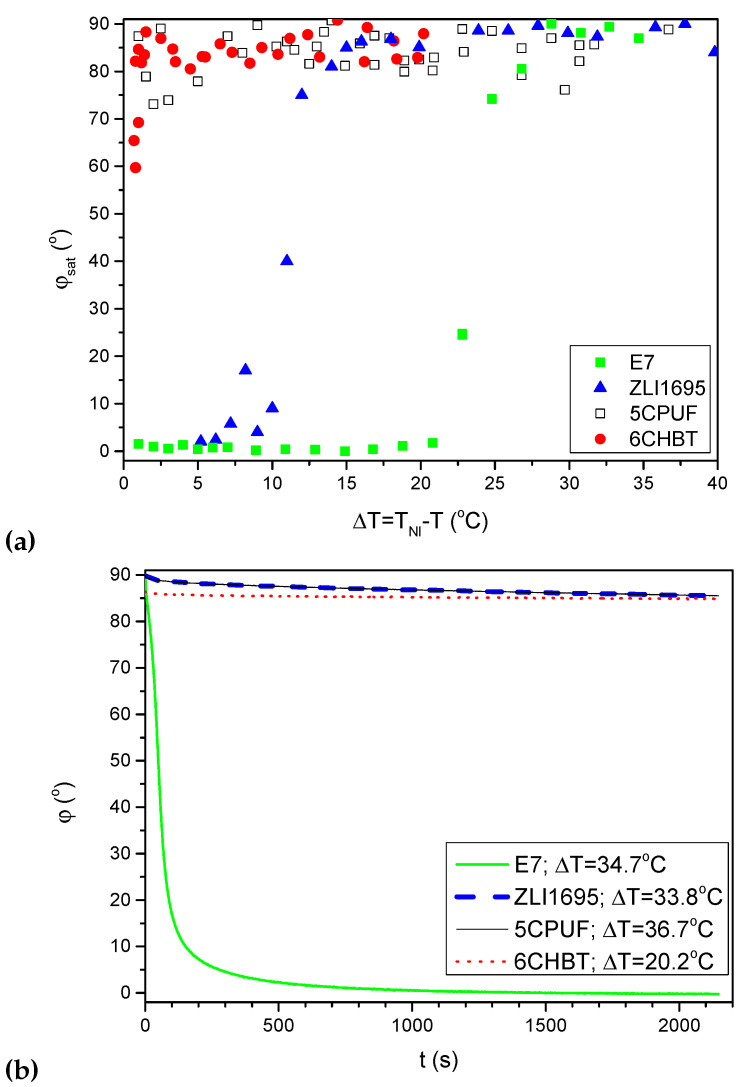
(**a**) Temperature dependence of the saturated azimuthal photoalignment angle, $\varphi_{sat}$ for nematics as indicated in the legend; (**b**) Temporal evolution of the back-relaxation of the azimuthal photoalignment angle at room temperature (pump beam switched off at t=0).

*Azimuthal photoalignment* induces a twist deformation in the cell with initial planar orientation: in the direct geometry, when the polarisation of the pump beam is parallel with the nematic director **n**, the efficient azimuthal photoalignment induces a saturation twist angle, $\varphi_{sat}$ with a maximum value of $90^{{}^{\circ}}$ at the photosensitive plate. Figure 3a shows the temperature dependence (relative to the nematic-to-isotropic phase transition temperature, $T_{NI}$) of the saturated photo-induced twist angle $\varphi_{sat}$, for some nematic liquid crystals as indicated in the legend.

In the temperature range far below the clearing point ($\Delta T=T-T_{NI}>25{\;}^{{}^{\circ}}$C), efficient azimuthal photo-orientation ($\varphi_{sat}\geq 80^{{}^{\circ}}$) was found for all nematics, regardless of the molecular structure (except for 5CB, which has $T_{NI}$ relatively close to the room temperature [21]). With the increase in temperature, however, the efficiency decreases. For cyano-biphenyl compounds (5CB, E7, E63), a drastic decrease in $\varphi_{sat}$ occurred far below $T_{NI}$ (at approximately ΔT<30–35 ${}^{{}^{\circ}}$C [21]), and $\varphi_{sat}$ practically drops down to zero in a wide nematic temperature range. For other compounds, such a decrease in $\varphi_{sat}$ is detectable much closer to $T_{NI}$, typically for $\Delta T<10{\;}^{{}^{\circ}}$C—see Figure 3a. 5CPUF with a fluorinated phenyl ring in the biphenyl rigid core belongs to this group: no considerable decrease in the efficiency of the azimuthal photo-orientation was detected in the temperature range of the nematic phase.

In the temperature range in which the efficiency of the azimuthal photoalignment decreases, it is straightforward to suppose that the light energy contributes to a *zenithal photoalignment*: such a photo-orientation also complies with the basic requirement that the redistribution of the azo dye moiety is perpendicular to the polarisation of the pump beam. Indeed, measurements with the light polarisation of the pump beam perpendicular to **n** (where no azimuthal photo-orientation is expected) have indicated a considerable zenithal photoalignment for nematics with cyano-biphenil rigid core (5CB, E7, E63) in the temperature range where the efficiency of the azimuthal photo-orientation has decreased [21]. In contrast, for other nematics (including 5CPUF), no such considerable zenithal photoalignment was detected at any temperature of the nematic phase [19,23]. Therefore, the decrease in the efficiency of the azimuthal photoalignment at high temperatures ($\Delta T<10{\;}^{{}^{\circ}}$C) in these compounds—see, e.g., ZLI1695 in Figure 3a—remained puzzling.

At high temperatures, just below $T_{NI}$, however, an increase in the pretilt angle, θ was also detected at the pDR1-LC interface, without any irradiation of the sample. The pretilt angle was estimated via electric field-induced Fréedericksz transition, and for example, in the case of E7, it was found that it changes from $\theta \approx 1.5^{{}^{\circ}}$ at low temperatures to $\theta \approx 80^{{}^{\circ}}$ at $\Delta T=2{\;}^{{}^{\circ}}$C, i.e., which is practically a temperature-induced anchoring transition from planar to homeotropic [21]. With this temperature-induced orientational transition, the decrease in $\varphi_{sat}$ at high temperatures—near $T_{NI}$—can be explained.

The back-relaxation from the azimuthal photoalignment was also monitored after the pump irradiation is switched off [19,23]. In general, the back-relaxation becomes faster with the increase in temperature. A distinct difference was also found which concerns the molecular structure of the nematics, and is illustrated in Figure 3b, which shows the temporal evolution of back-relaxation for various LCs at room temperature (pump irradiation switched off at t=0). Measurements have shown that at a given temperature, the back-relaxation of LCs with cyano-biphenyl rigid core (5CB, E7, E63 and 8CB) is faster by orders of magnitude than that of the other LCs (including 5CPUF).

Turning to the interpretation of the above results, we discuss the factors, which determine the saturation value of the light-induced twist. An obvious requirement to achieve complete azimuthal reorientation in a cell is that the light-induced azimuthal anchoring strength, *W*, should be sufficiently high to prevent the elastic back-relaxation of the nematic film. Quantitatively, this requires that the azimuthal extrapolation length, K/W (where *K* is the Frank elastic constant of the nematic), should be much smaller than the cell thickness. In addition, there is another process which can also lead to the back-relaxation of the induced twist deformation, namely the slow drift of the director on the LC−polymer interface, called *director gliding* [24,25,26,27,28,29]. The two processes can be distinguished from the speed by which the relaxation takes place. For anchoring effects, the relaxation time to the equilibrium state is in the order of $\gamma /KL^{2}$, where γ is the rotational viscosity of the nematic and *L* is the sample thickness—a few seconds in our cells. In the case of gliding, the relaxation time is much longer and it is usually non-exponential [29]. To obtain complete light-induced reorientation, it is necessary that no significant gliding occurs during irradiation.

For our materials, it was found that for the compounds, which have a phenylcyclohexane, or bicyclohexane rigid core, the saturation angle was almost $90^{{}^{\circ}}$, indicating that conditions for complete azimuthal reorientation were satisfied (except very near to the nematic–isotropic transition temperature). The same holds for the 5CPUF compound. Regarding the cyanobiphenyl compounds, we found that, although the back-relaxation is much faster than for the other LCs, it is still significantly slower than would be expected from an elastic relaxation. Therefore, we assert that the incomplete azimuthal reorientation observed at higher temperatures is mainly due to the increase in the gliding process in these materials.

The mechanism of gliding is not yet fully understood. In [29], it was demonstrated that gliding significantly increases as the glass temperature of the polymer layer is approached, suggesting that the mobility of the polymer chains is an essential factor in the process. On the other side, the glass temperature of PMMA is approximately $110{\;}^{{}^{\circ}}$C, which is much higher than a typical temperature at which our experiments were performed. From this circumstance, one can conclude that the interaction of the azo dye and the liquid crystal molecules is responsible for the back-relaxation observed for the cyanobiphenyl compounds. In [23], we suggested the formation of offset stacked π−π aromatic interactions between the biphenyl rigid core of LCs and the azo-benzene moiety of pDR1. This interaction imposes the *trans* form of the dye and LC molecules to align parallel to each other. In [23], it was conjectured that this effect can cause conformational changes in the surface polymer chains and can lead to the thermal rearrangements of the orientation of the azo dye; thus, the azimuthal back-relaxation of the director is facilitated. The π−π interaction is, however, not effective in the case of, e.g., 5CPUF (because of the MESP modified by fluorene atoms), which may explain the different behaviour found for this compound.

In the case of zenithal reorientation, observed for cyanobiphenyls, the relaxation time was also found to be much longer than the elastic time-constant, showing once more the importance of gliding. We can assume that due to the π−π interaction, the DR1 molecules which stick out from the polymer surface promote the homeotropic orientation of the LC at the interface. When this interaction becomes comparable with the one between the polymer bone and the LC (which stabilises the planar configuration), a pretilt appears. The decrease in the nematic order parameter may amplify this effect; thus, it becomes stronger as the nematic–isotropic phase transition is approached. The light-induced zenithal reorientation can be also explained by this model. Because of orientationally selective excitation, irradiation with plane-polarised light should enlarge the number of dye molecules perpendicularly oriented to the polymer layer. As a result, the strength of the “homeotropic” part of anchoring is amplified and the tilt angle of the LC increases.

Photo-orientation at the 8CB–pDR1 interface was also measured. 8CB also exhibits a smectic A phase below the nematic phase. In the smectic A phase, no measurable azimuthal nor zenithal photoalignment has been detected [19]. This is in accordance with expectations, since the bend and twist deformations in smectic A phase involve changes in the layer spacing requiring very high energy [30]. In the nematic phase, 8CB possesses similar photoalignment characteristics as the other cyanobiphenyl LC compounds discussed above, except slightly above the smectic A—nematic phase transition temperature, $T_{NA}$. In the high temperature range of the nematic phase, just below $T_{NI}$, no considerable azimuthal photoalignment and notable zenithal photo-orientation were detected, similarly to other cyanobiphenyls. With the decrease in the temperature, in a narrow temperature range, an incomplete azimuthal photoalignment (with $\varphi_{sat}\leq 75^{{}^{\circ}}$) and no considerable zenithal photo-orientation were found. With the further decrease in temperature, close to the smectic A phase (for $T_{NA}<T\leq T_{NA}+1{\;}^{{}^{\circ}}$C), both the azimuthal and the zenithal photoalignment vanished. This fact is presumably connected to the pretransitional fluctuations, which significantly increase the bend and twist elastic constants [31,32,33]. Due to the increase in these constants, the bulk elastic energy of the nematic film can decrease the effect of the surface anchoring, thus decreasing the efficiency of photoalignment.

## 4. Photoalignment in Different Phases of the Liquid Crystal

In this section, we show that the kinetics of photoalignment is not only influenced by the properties of the photosensitive film, but also by the state of order of the liquid-crystalline compound in contact with it [34]. The experiments were carried out with 8CB and 8OCB, which both have smectic A and nematic phases. The smectic A-nematic phase transitions occur at $32{\;}^{{}^{\circ}}$C and at $67{\;}^{{}^{\circ}}$C; the clearing points are at $39{\;}^{{}^{\circ}}$C and $80{\;}^{{}^{\circ}}$C for the two compounds, respectively.

For the investigations, we used as a photosensitive layer a polyimide film, in which Disperse Orange 3 was physically included (see Section 2). This substrate requires longer irradiation times to induce reorientation than the one with azo-functionalised polymers; on the other hand, the resulting photoalignment is much more stable than in the latter system, i.e., it does not show gliding.

In these experiments, the cells were filled in the isotropic phase and cooled to the smectic phase in the presence of a strong magnetic field, parallel to the rubbing direction. Photoalignment was induced with an unfocused argon laser beam at 488 nm with a power level of 70 mW. The polarisation direction of the pump beam was at $45^{{}^{\circ}}$ from the rubbing direction. The irradiation was carried out at different temperatures, corresponding to different phases of the liquid crystal, while the induced twist angle was measured at a fixed temperature, within the nematic phase of the liquid crystal.

**Figure 4:**
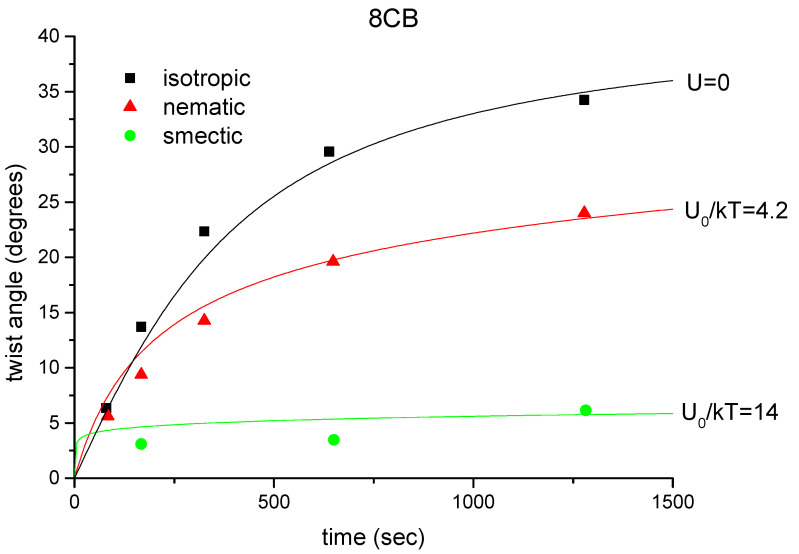
Induced twist as a function of time for irradiations in the different phases of 8CB. Symbols: experimental results. Continuous lines: theoretical fits with $C_{0}=2.1$ and $1/p_{0}=2500$ s (see text).

**Figure 5:**
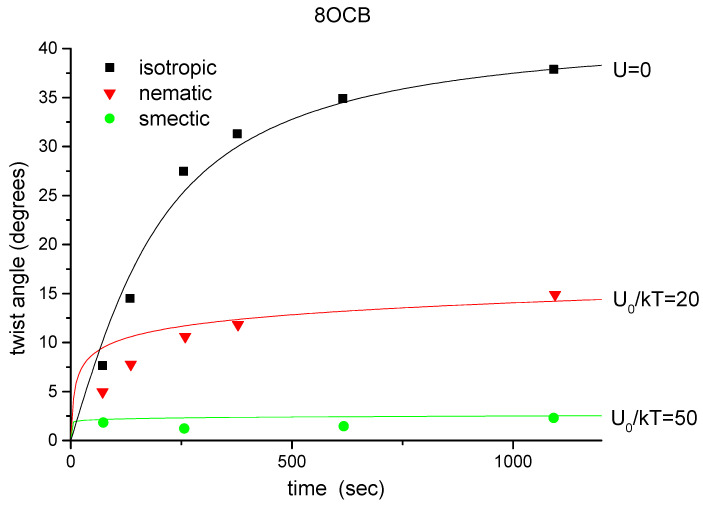
Induced twist as a function of time for irradiations in different phases of 8OCB. Symbols: experimental results. Continuous lines: theoretical fits with $C_{0}=2$ and $1/p_{0}=1360$ s (see text).

The experimental results are shown in Figure 4 and Figure 5. From the curves, one can clearly see the significance of the liquid-crystalline order in the kinetics of photoalignment. For both materials, the reorientation is most efficient when the irradiation takes place in the isotropic phase; less efficient, but still significant, when the liquid crystal is in the nematic phase; in the smectic phase, the induced twist angle is only a fraction of that obtained after the same irradiation in the isotropic phase. It is also evident that this effect cannot be attributed to the temperature difference of irradiation. We may compare the curves taken at $50{\;}^{{}^{\circ}}$C and $60{\;}^{{}^{\circ}}$C for 8CB and 8OCB, respectively. Although the temperature was even lower in the former case, the irradiation induced much higher twist angles when the polymer was in contact with the isotropic 8CB than when it was interacting with the smectic 8OCB.

**Figure 6:**
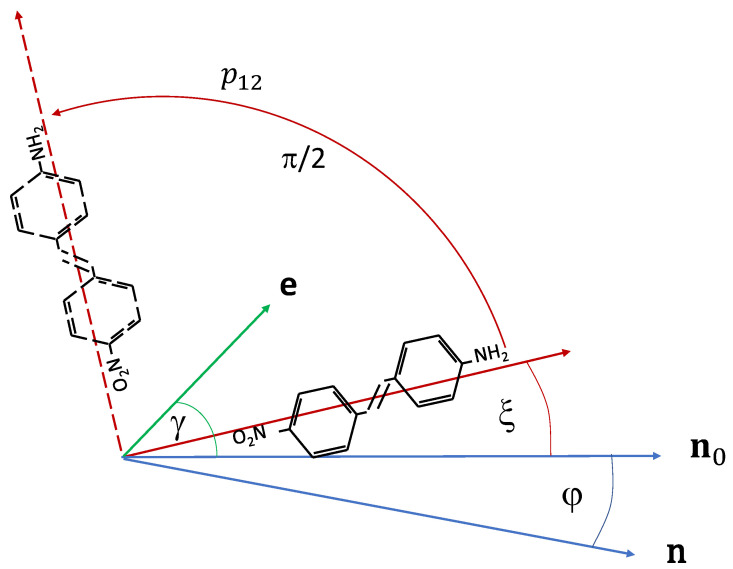
Sketch of the reorientation mechanism proposed in this model. A dye molecule, with original orientation along ξ, can change its orientation by 90° via *trans–cis–trans* cycle. $p_{12}$ is the transition probability, **n**_0_ is the original director on the polymer surface, **n** is the actual one. The angle between the light polarisation (**e**) and **n**_0_ is *γ*, which was 45° in our experiments. During the irradiation, in the smectic phase, the twist angle, *φ*, is assumed to be zero, while in the nematic phase, it continuously changes.

In the following, we describe a simple model to explain the above observations [26]. We suggest that the interaction between the liquid crystal and the azo dye molecules at the polymer–liquid crystal interface leads to a sort of guest–host effect, in the sense that the liquid crystal has an orienting effect on the dye molecules. For simplicity, we assume that every azo dye molecule has two stable orientations, which are perpendicular to each other, and the transition dipole moment is parallel to the orientation, as schematically shown in Figure 6. Transitions between the two stable orientations can be induced by light through *trans–cis–trans* cycle. The basic assumption of the model is that the transition probability from orientation 1 to 2 ($p_{12}$) and that of from 2 to 1 ($p_{21}$) differs by a Boltzmann factor exp(U/kT), where *U* is the difference of the orientational potential within the liquid crystal for the two perpendicular orientations. The transition probabilities of a switch of a dye molecule from the orientation ξ to ξ/2 and from ξ/2 to ξ, respectively, can be written as

(1) $$p_{12}=p_{0}e^{-\frac{U}{2kT}}\cos^{2}\left(\xi -\gamma \right);\;p_{21}=p_{0}e^{\frac{U}{2kT}}\sin^{2}\left(\xi -\gamma \right),$$

where $U=U_{0}\cos2\left(\xi -\varphi \right)$; φ is the actual azimuthal angle of the director on the photosensitive plate and γ is the angle of light polarisation with respect to the rubbing direction. The factors containing (ξ−γ) take account of the orientationally selective excitation probability. For the smectic and nematic phases, $U_{0}$ is of the same order of magnitude as the liquid crystal mean-field potential, i.e., it is a few times kT, while for the isotropic phase, it is zero.

The dye distribution function can be determined from a simple rate equation. Considering the dye molecules, which are allowed to align along ξ and ξ/2, we denote by x=x(ξ) the fraction of dye molecules, which are oriented along ξ. The rate equation reads:

(2) $$dx/dt=(x_{s}-x)/\tau$$

with $1/\tau =p_{12}+p_{21}$ and $x_{s}=p_{21}/\left(p_{12}+p_{21}\right)$.

As is discussed in [34], there are two contributions to the azimuthal anchoring energy of the liquid crystal on the photosensitive plate. The first one results from the interaction energy of the azo dye molecules with the liquid crystal; the second part is a permanent anchoring energy, independent from the photo-orientation process. In our experiment, the latter one is connected to the so-called “memory effect” [35] observed when a liquid crystalline material is cooled down from the isotropic phase in the presence of a magnetic field. The permanent anchoring initially stabilises the planar alignment.

The easy axis of the nematic on the photosensitive substrate (φ) is given as the minimum of the total anchoring energy. A simple consideration shows, assuming that the elastic twist energy of the nematic layer can be neglected, that φ can be calculated as

(3) $$\varphi =\frac{1}{2}\arctan\frac{S}{C+C_{0}},$$

where:

$$S=\int_{0}^{\pi }x\left(\xi \right)\sin2\xi d\xi ,\;C=\int_{0}^{\pi }x\left(\xi \right)\cos2\xi d\xi ,$$

and $C_{0}$ is the ratio of the permanent and dye-induced anchoring strengths.

From Equations (1)–(3), the twist angle can be calculated as a function of time. For the nematic phase, the actual director is recalculated after each time-step. For the smectic phase, φ is kept constant at its initial value, in accordance with the observation that, within this phase, there is no change of the director during irradiation (see Section 3). In the isotropic phase, the potential $U_{0}$ is zero.

In Figure 4 and Figure 5, we show the theoretical fits based on our model. The initial condition corresponds to an unoriented dye distribution, i.e., x(ξ)≡1/2. The parameter $C_{0}$ was deduced from the experimentally observed azimuthal reorientation induced in the isotropic phase. The parameter $U_{0}$ was estimated from the data obtained from the nematic and smectic phase irradiations, using the same value of $C_{0}$ as for the isotropic phase. We note that $U_{0}$ was independently chosen for the nematic and smectic phase irradiations, since the orienting potential is also different in the two phases: it is higher in the latter phase as in the former one [36].

The theoretical curves reveal obvious qualitative similarity to the experimental observations. Quantitatively, one can obtain reasonable agreement between the calculated curves and the measured data. However, the potential parameters used for the fits are much higher in the case of 8OCB than for 8CB. This fact also demonstrates the importance of the molecular structure of the liquid crystal in the process of photoalignment.

## 5. Conclusions and Outlook

In summary, the above results clearly indicate that the photoalignment efficiency does not solely depend on the composition of the photo-orienting layer, but also on the liquid crystal interfacing the layer. Azimuthal and zenithal photoalignments were found to be sensitive to the molecular structure of the LCs, as shown in the example of nematics having biphenyl, phenylcyclohexane and bicyclohexane in the rigid core. In addition, we demonstrated that photoalignment is also sensitive to the mesomorphic state of the LC in contact with the photosensitive layer, as it was proved on the example of 8CB and 8OCB having isotropic nematic and smectic A phases.

In the present communication, we showed the importance of director gliding in the relaxation process—both in azimuthal and zenithal reorientations. The extent of gliding was also found to be sensitive to the core structure of the liquid crystal, which indicates the importance of the interaction strength between dye and liquid crystal molecules. We attributed the observed differences to the π−π interaction between the DR1 and the nematic molecules. In the case of photoalignment in different phases of liquid crystals, the two compounds investigated showed qualitatively similar behaviour. However, in the theoretical fitting, we found a significant difference in the interaction potential for 8CB and 8OCB. This fact shows once again the sensitivity of the interaction between the azo dyes and the mesogens on the molecular structure of the liquid crystal.

There are, obviously, many open questions related to this subject, which could be resolved by further experiments and modelling. As an example, we can mention the more quantitative characterisation of the anchoring strength and gliding. The extension of the two-dimensional model presented in Section 4 to three dimensions may have significance related to the quantitative analysis of zenithal reorientation. It is also of interest to compare polymers containing functionalised azo dyes with the ones in which the dyes are only physically included.

Photoalignment can also be realised with substrates covered with a monolayer of azo dyes [37,38]. The question thus arises of whether can one expect similar effects with such systems as in the case of polymer substrates? The answer to this question should help to elucidate the role of the polymer network in photo-orientation.

## Data Availability

Not applicable.
